# Mechanical properties and fire resistance of composite bamboo materials heat-treated with MOS gelling adhesive at different temperatures

**DOI:** 10.1371/journal.pone.0350812

**Published:** 2026-06-17

**Authors:** Ximing Liu, Hongyun Cao, Jingfan Ma

**Affiliations:** 1 School of Communication and Design, Longyan University, Longyan, China; 2 School of Life Sciences, Longyan University, Longyan, China; Srinivas Institute of Technology, INDIA

## Abstract

Given the flammability and insufficient MPs of natural bamboo, this study uses Magnesium Oxysulfate (MOS)-Gelling Adhesive (MOS-GA) to prepare high-performance composite bamboo, and examines the effects of various temperature treatments on the MPs and fire resistance of MOS-GA composite bamboo. The results showed that MOS-GA had the best MPs after heat treatment at 400°C. After 7 days of curing, the CS of the specimen ranged from 90.02 MPa to 92.37 MPa, and the FS ranged from 9.52 MPa to 9.67 MPa. The MPs at 600°C decreased, the CS dropped to 10.17 MPa-18.24 MPa, and the FS dropped to 2.08 MPa-3.02 MPa. When treated with MOS-GA at 400°C, the composite bamboo material had a maximum along-grain CS of 79.36 MPa, and a TS and FS of 33.45 MPa and 72.12 MPa. The fire resistance limit of the specimen treated at 400°C reached 98 min, the charring depth was 35.2 mm at 100 min, the internal temperature distribution was reasonable, and it showed good thermal insulation performance and structural integrity. Research shows that moderate heat treatment temperature can effectively balance the MPs and fire resistance of MOS-based composite bamboo, providing a theoretical basis for its application in green fire-resistant buildings.

## 1. Overview

As a natural renewable biomass material, bamboo has the characteristics of short generation cycle, high specific strength, and excellent environmental performance. It has great application potential in green buildings and sustainable structural engineering [1,2]. However, natural bamboo has defects such as anisotropy, easy hygroscopic degradation, susceptibility to mildew, and flammability, which limit its application in high-performance structural components [3]. To overcome the above limitations, the preparation of a Composite Bamboo Material (CBM) with excellent Mechanical Properties (MPs) and Fire Resistance (FR) has become the key to current research in wood science and civil engineering [4]. CBM can effectively improve the physical MP of natural bamboo, and can also improve its durability and FR by introducing functional cementitious materials [5]. A large number of scholars have conducted relevant research on CBM and its performance.

G. Sheng et al. explored the impact of alkali treatment on the MP and water resistance of CBM. They used different concentrations of sodium hydroxide to treat CBM and characterized its structure and properties through Scanning Electron Microscopy (SEM) and X-ray Diffraction (XRD). 3wt% alkali treatment optimized the mechanical strength of the composite material and the softening coefficient reached 0.57, which effectively improved the interface adhesion and water resistance [6]. X. Mai et al. proposed a CBM with efficient anti-biofouling properties to address the problem that bamboo is susceptible to microbial corrosion. This study improved its antimicrobial pollution performance by adding chitosan to zinc oxide into a hydrophobic association hydrogel and filling it in-situ in the pores of bamboo to build a protective layer. This CBM had excellent antibacterial rate [7]. To address insufficient strength of CBM, S. Ochi explored the effect of random reinforcement of bamboo fiber bundles on its MP. This study prepared and tested materials by regulating the moisture content of bamboo powder, molding temperature, and fiber bundle content. At 180°C and the fiber bundle content was 70%, the Tensile Strength (TS) and Flexural Strength (FS) of CBM were optimal, reaching 45.0 and 101.4 MPa [8]. S. Han et al. discussed CBM and its structure-constructed MP, described the structural features of the bamboo wall layer along the thickness and height directions from the aspects of chemical composition, gradient structure, pore structure, variable cross-section hollow structure, etc., and developed new CBM. The study pointed out that the structural optimization of CBM provides important inspiration for the design of ultra-light and high-strength materials [9]. P. Barman et al. analyzed the CBM preparation methods of two molding technologies, compression and manual lamination, and explored their thermodynamic characteristics. This study used different concentrations of sodium hydroxide to treat bamboo, and combined mechanical testing and SEM to characterize it. The MPs of CBM prepared by compression molding were better than those of manual lamination [10]. S. R. Mousavi et al. explored the MP of bamboo fiber reinforced polymer composites by analyzing the characteristics of bamboo fiber, animal fiber and mineral fiber to improve the MP of composite materials. Bamboo fiber was an excellent reinforcing material, and modification of its surface could effectively improve the comprehensive MP of the composite main material [11]. Although the above-mentioned studies have analyzed the performance of CBM, most of them focus on organic polymer composite materials, which have shortcomings of insufficient high temperature resistance and poor long-term durability.

To further improve the high-temperature stability and FR of CBM, it is necessary to explore the characteristics of new Inorganic Gelling Materials (IGMs) and their application potential in CBM. As a durable and environmentally friendly binder, IGM can effectively improve the overall performance of composite materials [12]. Among many IGMs, Magnesium Oxysulfate (MOS) Bingding Material (MOS-BM) has received widespread attention since its advantages of rapid hardening and early strength, good wear resistance, strong adhesion, low production energy consumption, and small carbon footprint [13]. In recent years, scholars have conducted research on the preparation process and performance of MOS-BM. H. Huang and Q. Sun. proposed a performance optimization method for preparing MOS-BM using multi-source solid waste. This method improved performance by optimizing the particle size of wood powder and the synergistic blending ratio of fly ash, grinding slag, and water glass. After optimization, the Compressive Strength (CS) of the material reached 80MPa, the solid waste utilization rate reached 65%, and it had excellent MP and environmental benefits [14]. Aiming at the problems of slow condensation and insufficient strength of MOS, Y. Sun et al. explored the mechanism of improving the performance of MOS through ball milling modification of highly active magnesium oxide. This study used ball-milled light-burned magnesium oxide with an activity greater than 80% to mix with magnesium sulfate heptahydrate to accurately control the hydration reaction. The CS of the new MOS has been increased by 4 times and has excellent water resistance [15]. P. Hou et al. discussed the preparation of a new type of MOS-BM and analyzed its performance. This study systematically characterized its setting time, mechanical strength, water resistance, and microstructure by analyzing the reaction mechanism and products of magnesium oxide and different raw materials, combined with accelerated carbonization and organic hybridization technology. This new MOS-BM had the advantages of rapid hardening and early strength, high adhesion, and good water resistance [16]. N. Seyed Alireza et al. explored the reinforcement effect and performance of MOS, and analyzed the MP and microstructure of samples under different MOS dosages through triaxial testing, SEM and XRD. Increasing the MOS content could improve the CS and durability index of the sample [17].

Research has made certain progress in the preparation and performance of MOS-BM. However, existing research mostly focuses on the modification of the MOS material itself or its application in concrete and soil. As a process that effectively regulates the microstructure and final properties of materials, heat treatment has an unclear impact on the interface bonding state and stability between MOS-Gelling Adhesive (MOS-GA) and bamboo, as well as MP and FR [18]. In view of this, this study systematically explored the microstructure evolution rules of MOS-GA under different Temperature Heat Treatment (THT) conditions and its impact on the MP and FR of CBM. This study aims to prepare MOS-based CBM with excellent comprehensive properties through the optimization of heat treatment process, and provide experimental basis and theoretical support for its practical application in green buildings and FR structures. The innovation of this research lies in the successful development of a MOS-based CBM with both excellent MP and outstanding FR. By correlating the microstructural evolution of MOS-GA with macro mechanics and FR after different THT, it aims to clarify the optimal Heat Treatment Temperature (HTT) and achieve a synergistic improvement in the MP and FR limits of the material. This paper offers a new technical path and theoretical support for the development of new CBM with high load-bearing capacity and excellent fire safety.

## 2. Methodology

### 2.1. Experimental materials

The experimental materials are bamboo from a Bamboo Technology Co., Ltd. Bamboo is processed into bundles along the grain direction. Specific specifications and physical performance parameters: width is 10 mm ± 2 mm, thickness is 2 mm ± 0.2 mm, apparent density is 0.75 g/cm^3^. Before use, the bamboo bundles are placed in a blast drying oven at 60°C to dry to a constant weight, so that the moisture content is controlled below 8% for subsequent composite use.

### 2.2. Reagents

Table 1 shows the main experimental reagents used during the experiment.

**Table 1:** Information of experimental reagents.

Reagent Name	Purity	Manufacturers
Light-burned Magnesia (MgO)	Industrial Grade, Activity ≥ 85%	Liaoning Haicheng Magnesite Group Co., Ltd.
Magnesium Sulfate Heptahydrate (MgSO_4_·7H_2_O)	Analytical Reagent (AR), ≥ 99.0%	Sinopharm Chemical Reagent Co., Ltd.
Phosphoric Acid (H_3_PO_4_)	Analytical Reagent (AR), 85%	Aladdin Biochemical Technology Co., Ltd.
Sodium Silicate (Na_2_SiO_3_)	Modulus 3.3	Tianjin Komiou Chemical Reagent Co., Ltd.
Absolute Ethanol (C_2_H_5_OH)	Analytical Reagent (AR), ≥ 99.7%	Shanghai Macklin Biochemical Co., Ltd.
Deionized Water	/	Laboratory-made

### 2.3. Instruments

Table 2 shows the main instruments and equipment used.

**Table 2:** Information of instruments.

Name	Model	Manufacturer
Forced Air Drying Oven	DHG-9070A	Shanghai Yiheng Scientific Instrument Co., Ltd.
Electronic Balance	FA2004	Shanghai Shunyu Hengping Scientific Instrument Co., Ltd.
SEM	SU8010	Hitachi, Japan
XRD	D8 Advance	Bruker, Germany
Box-type Resistance Furnace	SX2-4-10	Shanghai Laboratory Electric Furnace Factory
Electronic Universal Testing Machine	CMT6104	MTS Industrial Systems (China) Co., Ltd.
Microcomputer Controlled Fully Automatic Pressure Testing Machine	SHT4605	Shanghai Xinsansi Metrology Instrument Manufacturing Co., Ltd
Constant Temperature Water Bath (CTWB)	HH-4	Changzhou Guohua Electric Appliance Co., Ltd.
Digital Temperature Recorder	TR-92A	Azbil Instrument (Taiwan) Co., Ltd.

### 2.4. Preparation method of MOS-GA

MgO and MgSO_4_·7H_2_O were utilized as the main raw materials, supplemented by H_3_PO_4_ and Na_2_SiO_3_ as modifiers, to prepare MOS-GA. Specific preparation method: First, MgO was dried at 105°C for 2 h to remove adsorbed moisture and improve reaction activity. According to the previous experimental results, the molar ratio of MgO to MgSO_4_·7H_2_O was set to 5:1, and the water-binder ratio (W/B) of MgO in the water area was 0.28. Then, MgSO_4_·7H_2_O was dissolved in dehydrated ions to prepare a magnesium sulfate (MgSO_4_) solution with a concentration of 25w%, and H_3_PO_4_ with 1% of the total MgO mass was added as a retarder and water resistance modifier. The pretreated MgO powder was slowly added to the MgSO_4_ solution, and mixed at a stirring speed of 500 r/min for 5 min. Then, 0.5w% Na_2_SiO_3_ was added as a dispersant, and stirring was continued for 10 min until the slurry was uniform. The evenly mixed MOS slurry was injected into a 40 mm × 40 mm × 160 mm triple mold, vibrated and exhausted, then placed at a standard curing condition temperature of 20 ± 2°C, and left to stand for 24 hours in a relative humidity ≥ 95% to demould. Subsequently, it was transferred to a CTWB at 20°C for curing to the specified age. To explore the effect of heat treatment on the performance of MOS-GA, this study placed the specimens cured for 7 days and 28 days in a box-type resistance furnace and heated them to 200, 400, 600, 800, and 1000°C, respectively at a heating rate of 5°C/min. The specimen was kept warm for 2 hours and then cooled to room temperature in the furnace to obtain MOS specimens with different THT.

The activity of MgO is a key factor affecting the final performance of MOS-BM, and its active ingredient content directly determines the degree of reaction with magnesium sulfate and the product structure [19]. The calculation of its active MgO content is shown in formula (1).


$$\begin{array}{c} W_{A}=((W_{b}-W_{a})/0.45W_{a})\times 100\% \end{array}$$ (1)

In formula (1), $W_{A}$ is the active MgO content (%). $W_{b}$ and $W_{a}$ are the mass (g) of the sample after drying with water and before drying. W/B is the core parameter that determines the rheology, compactness and final hardened body pore structure of the cementitious material slurry [20]. Its calculation is shown in formula (2).


$$\begin{array}{c} W/B=(m_{w}/(m_{\text{MgO}}+m_{{\text{MgSO}}_{\text{4}}})) \end{array}$$ (2)

In formula (2), $m_{w}$ denotes the water mass (g). $m_{\text{Mgo}}$ and $m_{{\text{MgSO}}_{\text{4}}}$ are the masses (g) of MgO and MgSO_4_.

### 2.5. CBM specimen design based on MOS-GA

To explore the effects of MP and FR of MOS-GA-based CBM under different THT conditions, two types of CBM specimens were designed in this study. All specimens used bamboo bundles along the grain direction as reinforcing materials, with MOS-GA as the matrix, and were prepared through the molding process.

**Fig 1:**
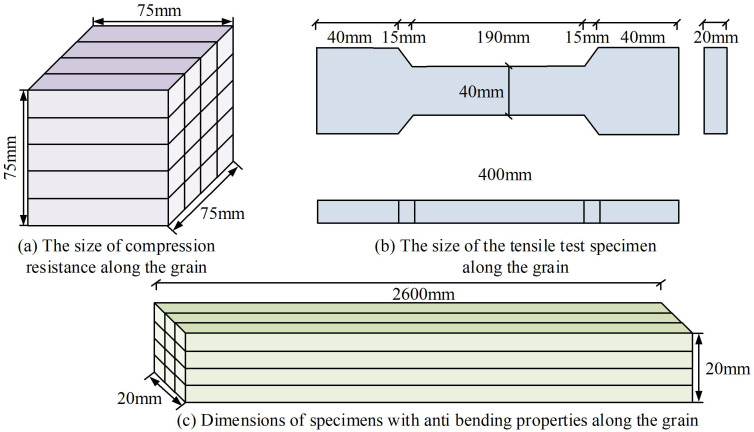
Dimensions of CBMs with MOS-GA for CS, TS, and FS along the grain.

MP specimens are mainly used to test the CS, TS, and FS of CBM in the along-grain direction [21]. The specimen size and preparation were designed according to ASTM D143 [22]. The dimensions of the three specimens of CBM compression along the grain are 75 mm × 75 mm × 75 mm. The three specimens were marked as M-Y-1, M-Y-2, and M-Y-3. For the three CBM tensile specimens (M-L-1, M-L-2 and M-L-3), the dimensions were 400 mm in length, 70 mm in side width, 40 mm in middle width, and 20 mm in thickness. The size of the CBM along-grain flexural specimens (M-Z-1, M-Z-2 and M-Z-3) was 2600 mm × 20 mm × 20 mm. The HTTs of MOS-GA were 200, 400, and 600°C. All MP specimens were cured under standard curing conditions for 28 days, followed by heat treatment, and finally subjected to MP testing. The size design of CBM along-grain compression, tensile, and flexural resistance specimens based on MOS-GA is shown in Fig 1.

**Fig 2:**
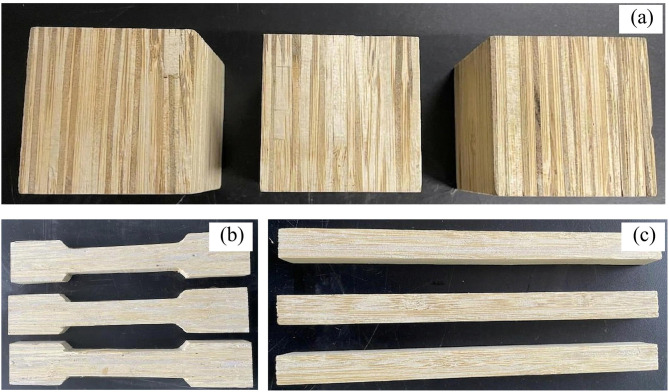
Physical photos of composite bamboo materials based on MOS-GA.

Fig 1(a) shows a cubic specimen for testing along-grain CS, with a design size of 75 mm × 75 mm × 75 mm. Fig 1(b) shows the specimen used for the along-grain TS test, with a total length of 400 mm. The width at both ends is 70 mm and is used for clamping. The width of the middle test section is 40 mm and the thickness is 20 mm. Fig 1(c) shows a long strip beam specimen used for along-grain FS testing, with dimensions of 2600 mm × 20 mm × 20 mm. The physical photos of composite bamboo materials based on MOS-GA are shown in Fig 2, including composite bamboo specimens for testing the compressive strength along the grain (a), tensile strength along the grain (b), and flexural strength along the grain (c).

**Fig 3:**
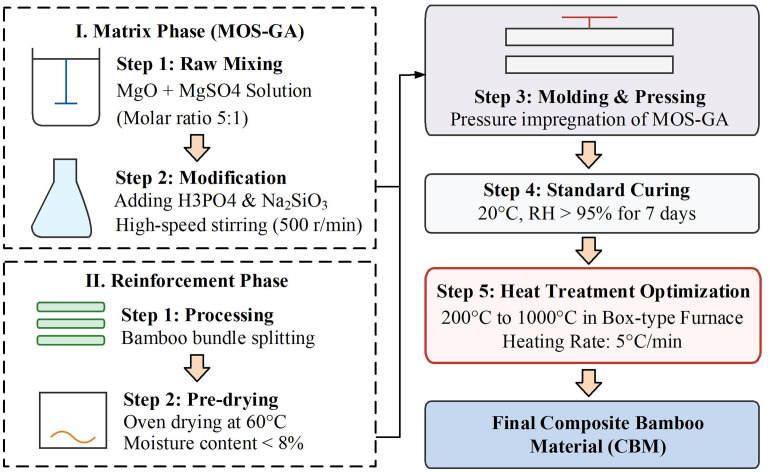
Synthesis of MOS-GA based composite bamboo material.

The FR specimen of CBM is mainly used to evaluate the FR limit, carbonization depth and internal temperature distribution of CBM at high temperatures [23]. The specimen design refers to “GB/T 9978-2008 Fire resistance test method for building components” [24]. In this study, three specimens are tested, designated as F-1, F-2, and F-3. The HTT of MOS-GA in the three specimens is all 400°C. The size of F-1 is 2100 mm × 120 mm × 160 mm, the span-to-height ratio is 10, and the load-carrying ratio is 20. The size of F-2 is 2100 mm × 120 mm × 160 mm, and the span-to-height ratio and load-carrying ratio are 10 and 30. The size of F-3 is 2500 mm × 140 mm × 220 mm, with a span-to-height ratio of 10 and a load-carrying ratio of 20. The schematic diagram of composite bamboo synthesis based on MOS-GA is shown in Fig 3.

In Fig 3, in the preparation of the matrix, lightly burned magnesium oxide and magnesium sulfate solution were mixed in a molar ratio of 5:1, and phosphoric acid and sodium silicate modifiers were added for high-speed stirring. Bamboo bundles are sliced and pre dried at 60 ° C until the moisture content is below 8%. Subsequently, MOS-GA was compounded with bamboo bundles through pressure impregnation and cured for 7 days at a standard temperature of 20 ° C and a relative humidity of over 95%. Finally, heat treatment optimization was carried out in a box type resistance furnace with a temperature increase of 5 ° C/min to 200 ° C-1000 ° C, resulting in the final production of composite bamboo.

### 2.6. Microstructure characterization and MP testing method of MOS-GA

#### 2.6.1. SEM analysis.

In this study, SU8010 SEM was used to analyze the micromorphology of MOS-GA [25]. After the sample was dried and sprayed with gold, it was placed on the SEM sample stage, and images were collected under the conditions of an accelerating voltage of 15 kV and a working distance of 8 mm [26]. The pore structure and crystal morphology of MOS-GA were analyzed through electron microscope images. The calculation of its porosity (%) P is shown in formula (3).


$$\begin{array}{c} P=(A_{p}/A_{t})\times 100\% \end{array}$$ (3)

In formula (3), $A_{p}$ is the total pore area (μm^2^). $A_{t}$ is the total analysis area of the image (μm^2^). The average pore diameter (μm) $D_{\text{avg}}$ is calculated as shown in formula (4).


$$\begin{array}{c} D_{\text{avg}}=\left(\overset{n}{\underset{i=1}{\sum }}d_{i}\right)/n \end{array}$$ (4)

In formula (4), $d_{i}$ is the equivalent diameter (μm) of the i-th pore. n is the total number of pores.

#### 2.6.2. XRD analysis.

This study used a Bruker D8 Advance XRD analyzer to analyze the phase composition and crystal structure changes of MOS-GA [27]. Test conditions: Cu-Kα radiation (λ = 1.5406), voltage 40 kV, current 40 mA, scanning range 2θ = 5°–70°, scanning step size 0.02°, and dwell time per step 0.5 s [28]. The samples were ground into powder and pressed into tablets for sample preparation. In this study, Jade software was used to identify the phases of the diffraction patterns, and the Rietveld full spectrum fitting method was utilized to calculate the content of each phase. The mass fraction of each phase is calculated as shown in formula (5).


$$\begin{array}{c} W_{j}=\frac{I_{j}\cdot K_{j}}{\overset{m}{\underset{j=1}{\sum }}(I_{j}\cdot K_{j})}\times 100\% \end{array}$$ (5)

In formula (5), $W_{j}$, $I_{j}$, and $K_{j}$ are the mass fraction (%), characteristic peak intensity, and reference intensity ratio of phase j. m is the gross of physical phases. In addition, the crystallinity of the main hydration products in MOS-GA is calculated based on the XRD analysis pattern, as shown in formula (6).


$$\begin{array}{c} C_{r}=\left(\sum I_{\text{crystalline}}/\left(\sum I_{\text{crystalline}}+\sum I_{\text{amorphous}}\right)\right)\times 100\% \end{array}$$ (6)

In formula (6), $C_{r}$ is the crystallinity. $I_{\text{crystalline}}$ is the sum of the diffraction peak intensities of the crystal phase. $I_{\text{amorphous}}$ is the sum of the amorphous phase scattering intensities.

#### 2.6.3. MOS-GA’s MP test method.

To evaluate the performance of MOS-GA as a CBM connection material, its cured MP needs to be tested, including FS, CS, and the Ignition Loss Rate (ILR) of the sample. The FS of MOS-GA is tested using an automatic flexural and compressive testing instrument, as shown in formula (7) [29].


$$\begin{array}{c} f_{f}=3F_{f}l/2bh^{\text{2}} \end{array}$$ (7)

In formula (7), $f_{f}$ represents FS (MPa). $F_{f}$ is the maximum load (N) at which the specimen breaks. l is the span between support rollers (mm). b and h are the cross-sectional width (mm) and height (mm) of the specimen. Then, in this study, the CS test was performed on the specimens that broke after the FS test, as shown in formula (8).


$$\begin{array}{c} f_{c}=F_{c}/A \end{array}$$ (8)

In formula (8), $f_{c}$ represents CS (MPa). $F_{c}$ means the maximum pressure load (N) when the specimen fails. A is the pressure-bearing area of the specimen (mm^2^). To evaluate the stability of MOS-GA in high-temperature environments, this study conducts an ILR test on it. In this method, a sample of a specified shape is baked to a constant weight at 105°C, then placed in a high-temperature furnace and burned at a set temperature, and the ILR is calculated based on the mass change before and after burning, as shown in formula (9).


$$\begin{array}{c} LOI=((m_{\text{0}}-m_{\text{1}})/m_{\text{0}})\times 100\% \end{array}$$ (9)

In formula (9), LOI represents ILR (%). $m_{\text{0}}$ and $m_{\text{1}}$ are the dry mass (g) of the sample before and after burning. This study uses three MOS specimens for testing, marked as J-1, J-2, and J-3.

### 2.7. CBM’s MP test method

To explore the MP of MOS-GA-based CBM, this study tested its along-grain CS, TS, FS, and Quality Loss Rate (QLR). All tests were conducted in a standard laboratory environment. Each group of specimens was tested three times, and the mean was taken as the final result.

In the along-grain CS test of CBM, when the specimen finally fails under the action of continuously increasing axial pressure, the ultimate pressure load it can withstand is the key indicator to measure its ability to resist compressive deformation [30]. The calculation of the grain-following CS of the proposed CBM is shown in formula (10).


$$\begin{array}{c} L_{fc}=L_{Fc}/(s_{w}\cdot s_{t}) \end{array}$$ (10)

In formula (10), $L_{fc}$ is CS (MPa) along the grain. $L_{Fc}$ is the maximum load (N) of the CBM specimen under compression. $s_{w}$ and $s_{t}$ are the width (mm) and thickness (mm) of the specimen. When CBM is subjected to axial tensile load, stress will be generated internally to resist separation, and the maximum value of this stress determines the tensile load-bearing capacity of the material. The calculation of TS along the grain is shown in formula (11).


$$\begin{array}{c} L_{fl}=L_{Fl}/(s_{w}\cdot s_{t}) \end{array}$$ (11)

In formula (11), $L_{fl}$ is along the grain TS (MPa). $L_{Fl}$ is the maximum load (N) of the CBM specimen in tension. Under the action of bending moment, the stress distribution on the specimen section is uneven. The maximum tensile stress occurs at the outermost fiber of the tensile surface. When this stress reaches the ultimate strength of the material, it will cause fracture. The calculation of its along-grain FS is shown in formula (12).


$$\begin{array}{c} L_{ff}=3L_{Ff}l/2bh^{\text{2}} \end{array}$$ (12)

In formula (12), $L_{ff}$ is along the grain FS (MPa). $L_{Ff}$ is the maximum load (N) when the CBM specimen breaks. QLR is a key parameter characterizing the thermal stability of materials in high temperature environments. This study calculated the QLR by comparing the mass difference of the samples before and after heat treatment.

### 2.8. CBM’s FR test method

**Fig 4:**
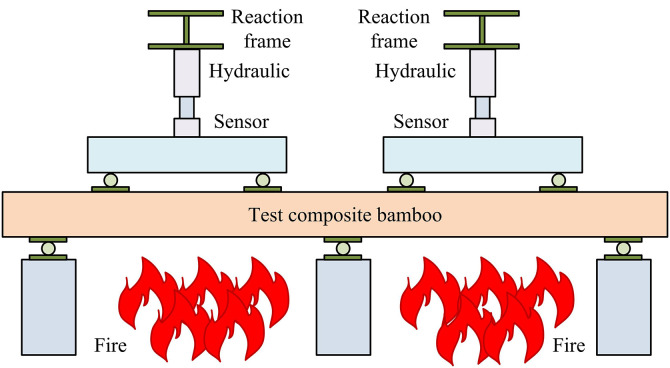
Fire simulation diagram of composite bamboo.

To evaluate the FR of CBM based on MOS-GA, this study tested its FR limit, charring depth, and charring rate, as well as the internal temperature distribution of the specimen, aiming to explore the structural integrity and thermal insulation performance of the proposed CBM in a fire environment. The fire simulation diagram of CBM is shown in Fig 4.

In Fig 4, the experimental device mainly has a reaction frame, a hydraulic loading system, sensors and the CBM specimen to be tested. This setup simulates a real fire scenario, applying load to the specimen through the hydraulic system, and using sensors to monitor its deformation, internal temperature, structural integrity, and other key parameters at high temperatures in real-time to scientifically evaluate its fire resistance limit and thermal insulation performance. The FR limit of CBM is tested by monitoring the deformation of the test piece during the fire process. When the test piece reaches any of the following limit states, it is considered to have reached the FR limit, as shown in formula (13).


$$\begin{array}{c} \left\{ \begin{array}{c} @l@d=B^{\text{2}}/400q\\ \frac{qd}{dt}=B^{\text{2}}/9000q \end{array}\right. \end{array}$$ (13)

In formula (13), d is the ultimate bending deformation of the CBM specimen (mm). q is the cross-sectional height of the specimen (mm). B is the clear span of the CBM specimen against bending (mm). After the test, the cooled specimen was sectioned in a direction perpendicular to the fire-receiving surface. At least 5 measurement points were evenly selected on the cross-section, and a digital depth gauge was used to accurately measure the vertical distance from the fire-receiving surface to the obvious charring dividing line. The calculation of the carbonization depth and carbonization rate is shown in formula (14).


$$\begin{array}{c} \left\{ \begin{array}{c} @l@d_{c}=\left(\overset{U}{\underset{u=1}{\sum }}d_{u}\right)/U\\ R_{c}=d_{c}/t_{f} \end{array}\right. \end{array}$$ (14)

In formula (14), $d_{c}$ is the average carbonization depth (mm). $d_{u}$ is the carbonization depth (mm) of the u-th measurement point. U is the total number of measurement points. $R_{c}$ is the carbonization rate (mm/min). $t_{f}$ is the FR limit (min). The internal temperature gradient of the cross-section is a key indicator to characterize the thermal insulation performance of the material, reflecting the temperature drop per unit thickness of the material, as shown in formula (15).


$$\begin{array}{c} G=(T_{s}-T_{d})/x_{d} \end{array}$$ (15)

In formula (15), G is the internal temperature of the section (°C). $T_{s}$ and $T_{d}$ are the theoretical temperature (°C) and the measured temperature (°C) of the fire surface of the specimen. $x_{d}$ is the vertical distance (mm) between the temperature measuring point and the fire surface.

## 3. Results and analysis

### 3.1. Analysis of MP results of MOS-GA under different THT

**Fig 5:**
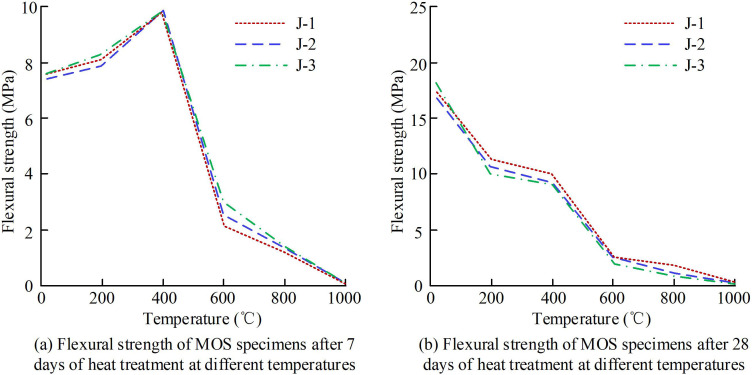
FS of MOS specimens after heat treatment at various temperatures.

To evaluate the effect of HTT on the MP of MOS-GA, this study tested and analyzed the FS, CS, and ILR of MOS specimens when the curing time was 7 days (7d) and 28 days (28d). Fig 5 shows the changes in specimen FS. In Fig 5(a), when the curing time was 7d, the FS of the MOS specimen (J-1) at a temperature of 400°C was 9.52 MPa, and the FS of specimens J-2 and J-3 were 9.67 MPa and 9.58 MPa. When the HTT reached 600°C, the FS of specimens J-1, J-2, and J-3 were 2.08 MPa, 2.67 MPa, and 3.02 MPa. In Fig 5(b), when the curing time was 28d, the FS of the three MOS specimens at the temperature of 400°C were 10.02 MPa, 9.10 MPa, and 8.75MPa, and the FS at the temperature of 600°C were 2.25 MPa, 2.23 MPa, and 2.06MPa. When the HTT was 400°C, the FS of MOS-GA reached the optimum.

**Fig 6:**
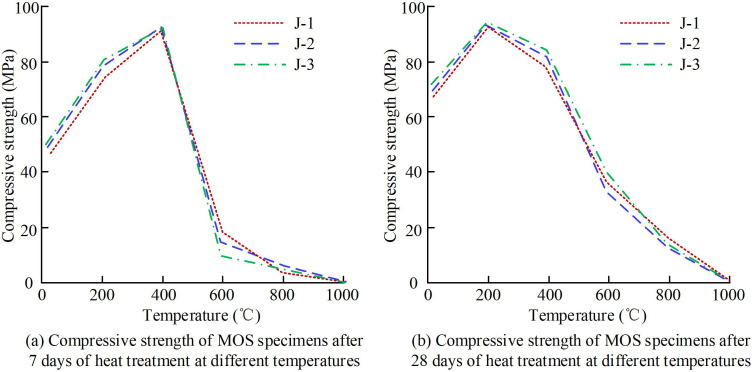
CS of MOS specimens after heat treatment at various temperatures.

This study further tested and analyzed the MOS specimen CS 7d and 28d after different THT, as shown in Fig 6. In Fig 6(a), at 7d, the CS of MOS specimens J-1 and J-2 at 400°C were 90.02 MPa and 92.37 MPa, and the CS of specimen J-3 was 91.28 MPa. When increasing to 600°C, the CS of the three MOS specimens were 18.24 MPa, 16.53 MPa, and 10.17 MPa. In Fig 6(b), at 28d, the CS of MOS specimens J-1, J-2, and J-3 at 400°C were 78.24 MPa, 82.36 MPa, and 85.15 MPa, and the CS at 600°C were 36.27 MPa, 32.16 MPa, and 39.35 MPa. This study observed that the compressive strength of MOS adhesive after 28 days of heat treatment at 400°C was lower than that after 7 days. The fundamental reason for this is that the heat treatment was applied during the intermediate stage of hydration reaction (7 days), rather than standard curing until 28 days before heat treatment. Notably, Gu et al. [31] reported that while 400°C heat treatment can densify the microstructure of MOS cement and yield a high compressive strength of up to 133.8 MPa, the strength gain is highly dependent on the maturity of the hydration products prior to heat exposure. Their findings indicate that the 5·1·7 phase, which progressively develops between 7 and 28 days of standard curing, is susceptible to thermal decomposition when heat-treated at an early age, thereby compromising the late-stage strength development [31]. This corroborates the present observation that the compressive strength of MOS adhesive heat-treated at 400°C for 28 days was lower than that at 7 days, as the interrupted hydration process prevented the full formation of the 5·1·7 crystalline network.

**Fig 7:**
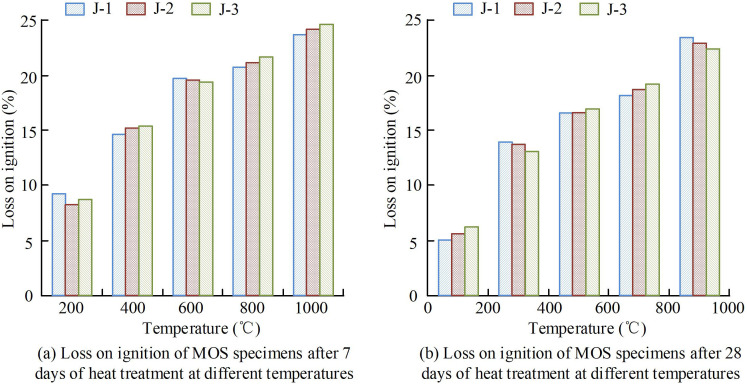
ILR of MOS specimens after heat treatment at different temperatures.

To explore the impact of different THT on the thermal mass loss of MOS-GA, this study tested the ILR of MOS specimens 7d and 28d after different THT, as shown in Fig 7. In Fig 7(a), at 7d, the ILRs of MOS specimens J-1, J-2, and J-3 at a HTT of 200°C were 9.12%, 8.36%, and 8.78%. When reaching 600°C, the ILR of the three specimens increased to 19.26%, 18.95%, and 18.27%. In Fig 7(b), at 28d, the ILRs of the three MOS specimens at 400°C were 14.23%, 13.98%, and 12.75%, and the ILRs at 600°C increased to 16.22%, 16.25%, and 17.15%. The increase in ILR directly reflects the degree of thermal decomposition of the 5·1·7 phase (5 Mg(OH)_2_·MgSO_4_·7H_2_O) and brucite (Mg(OH)_2_). These hydration products are the primary contributors to the mechanical strength of MOS cementitious materials. Their decomposition leads to a reduction in the effective load-bearing phase, which manifests as a sharp decline in mechanical strength. This loss of mechanical strength subsequently translates into reduced load-bearing capacity of the composite bamboo component when subjected to sustained fire exposure and applied service loads.

In this study, when the heat treatment temperature increased from 400°C to 600°C, the ILR increased from approximately 13%−14% to approximately 16%−19%, while the compressive strength decreased from approximately 90 MPa to 10–18 MPa, representing an 80%−89% loss rate. This drastic reduction in mechanical strength directly indicates that the material can no longer support its intended loads without excessive deformation or failure under fire conditions, i.e., the theoretical load-bearing capacity of the material is compromised. For the actual composite bamboo component, this strength degradation would manifest as a significant reduction in its fire resistance limit. This showed that after the HTT exceeded 400°C, the mass loss of MOS-GA increased significantly, and a longer curing time could form a more stable structure, which helps to improve the thermal stability of MOS.

### 3.2. Microstructure analysis of MOS-GA

**Fig 8:**
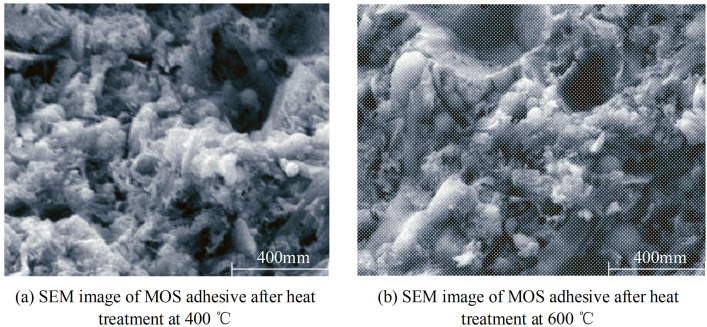
SEM image of MOS-GA after temperature heat treatment.

To explore the impact of different THT on the performance of MOS-GA, this study analyzed its microscopic star veins and phase composition through SEM and XRD. MP analysis of MOS-GA showed that the FS and CS of MOS-GA decreased significantly from 400°C to 600°C. Therefore, this study analyzed the SEM of MOS-GA after THT at 400°C and 600°C, as displayed in Fig 8. In Fig 8(a), after heating at 400°C, the microstructure of MOS-GA was relatively dense, mainly showing crystals after the decomposition of hydration products. These crystals were intertwined to form a continuous spatial network structure with good structural integrity. In Fig 8(b), after heating at 600°C, the microstructure of MOS-GA was destroyed, the hydration products decomposed in large quantities, the crystals transformed into loose, amorphous agglomerated, and obvious cracks were formed. After the HTT of MOS-GA was 600°C, the hydration product decomposed completely and its strength decreased. After heat treatment at 600 ° C, quantitative analysis of SEM images showed that the porosity increased from 12.3% at room temperature to 38.5%. The relative connected pore area ratio of the pore structure was calculated using image processing, and this ratio increased by about 2.3 times compared to room temperature at 600 ° C, indicating that the pores evolved from isolated distribution to a highly connected network. The connected pores caused the stress concentration factor to increase from 2.1 at room temperature to 5.4. Combined with DIC strain field analysis, the strain localization coefficient increased from 0.12 to 0.38, confirming that the highly connected pore network significantly intensified stress concentration and crack initiation, leading to a sharp decrease in compressive strength and flexural strength.

**Fig 9:**
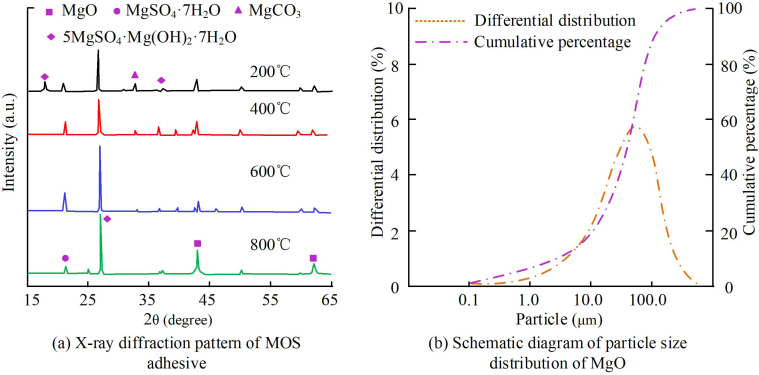
XRD pattern of MOS-GA and particle size distribution of MgO.

Fig 9 is an analysis of the XRD patterns of MOS-GA after different THT and the investigated particle size distribution results of MgO. In Fig 9(a), after heat treatment of MOS-GA, the main crystalline phase was 5·1·7 phase and a small amount of Mg(OH)_2_. When the HTT rose to 600°C, the intensity of the characteristic peaks of the 5·1·7 phase weakened, while the diffraction peaks of MgO and MgSO_4_ were significantly enhanced. This indicated that the main gelling hydration products have undergone thermal decomposition and converted into oxides and sulfates that are resistant to high temperatures but have no gelling activity. In Fig 9(b), MgO particles presented a unimodal distribution, and were mainly distributed around 30 μm. This relatively fine and concentrated particle size distribution was conducive to full contact and reaction with water and MgSO₄ solution in the early stages of preparation to form more hydration products, thereby ensuring the initial MP of MOS-GA. It was observed that sharp mechanical degradation above 400°C is primarily attributed to the complete thermal decomposition of the 5·1·7 phase (3 Mg(OH)_2_·MgSO_4_·7H_2_O) into MgO and MgSO_4_, accompanied by the loss of crystalline water and hydroxyl groups. This decomposition, confirmed by the disappearance of characteristic 5·1·7 peaks and the emergence of MgO peaks in XRD patterns, leads to a sudden collapse of the spatial network structure, as shown in SEM images. Unlike gradual strength loss caused by pore coarsening or microcracking, this abrupt phase transformation causes irreversible structural embrittlement and a drastic drop in both compressive and flexural strength.

**Fig 10:**
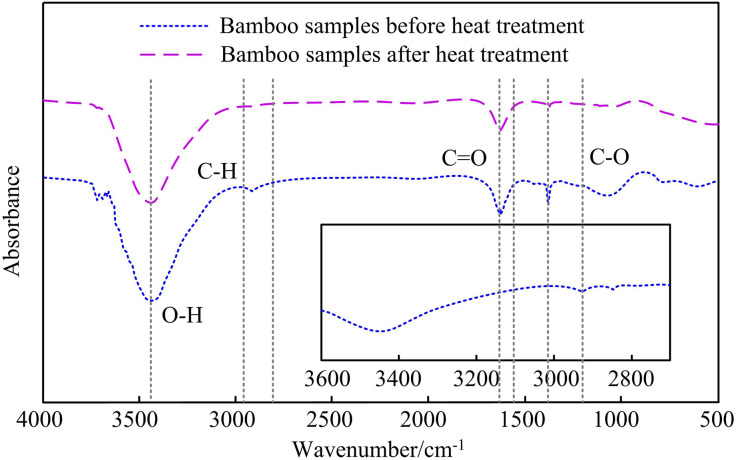
FTIR analysis of composite bamboo samples before and after heat treatment.

Further analysis was conducted on the Fourier Transform Infrared Spectroscopy (FTIR) spectra of composite bamboo samples before and after heat treatment, as shown in Fig 10. In Fig 10, untreated bamboo exhibits the following characteristic peaks: 3400 cm^-1^ (O-H stretching vibration of hydroxyl groups in cellulose and hemicellulose), 2920 cm^-1^ (C-H stretching vibration), 1735 cm^-1^ (C = O stretching vibration of acetyl groups in hemicellulose), and 1240 cm^-1^ (C-O stretching vibration of lignin). After heat treatment at 400 ° C, the peak intensities at 3400 cm^-1^ and 1735 cm^-1^ significantly decreased, indicating thermal decomposition of hemicellulose and partial degradation of cellulose. The peak at 1240 cm^-1^ also weakened, indicating a change in lignin structure. These FTIR results confirm that the 400 ° C heat treatment resulted in partial removal of thermally unstable components and altered the chemical environment of the bamboo surface, which may help improve the interfacial compatibility between bamboo and MOS adhesive. This discovery supports the performance of the prepared composite bamboo in terms of mechanical properties and fire resistance.

### 3.3. MP result analysis of CBM

**Fig 11:**
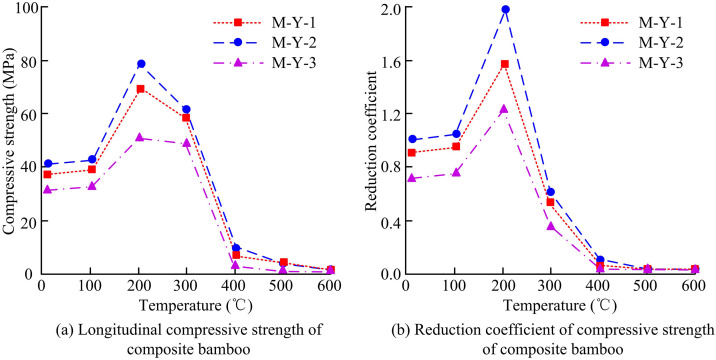
The longitudinal CS and reduction coefficient of composite bamboo.

To evaluate the impact of MOS-GA after different THT on the MP of CBM, this study conducted tests and analyzes on CBM specimens after different THT along the grain CS, along the grain TS, along the grain FS and QLR. This study first analyzed the along-grain CS and reduction coefficients of M-Y-1, M-Y-2 and M-Y-3 of CBM, as shown in Fig 11. In Fig 11(a), when the temperature was 200°C, the CS along the grain of M-Y-1, M-Y-2, and M-Y-3 were 68.27 MPa, 79.36 MPa, and 50.14 MPa. When increasing to 300°C, the CS of the three specimens along the grain decreased to 59.85 MPa, 60.57 MPa, and 48.56 MPa. In Fig 11(b), at 200°C, the CS reduction coefficients of M-Y-1, M-Y-2, and M-Y-3 are 1.52, 1.96, and 1.22, and at 300°C they were 0.55, 0.62, and 0.32. As the HTT of MOS-GA increased, the CS of CBM along the grain decreased, and the CS of specimen M-Y-2 was the best.

**Fig 12:**
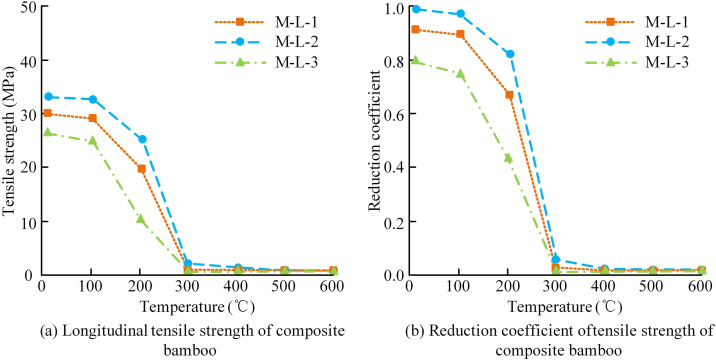
The TS along the grain and its reduction coefficient of composite bamboo.

Fig 12 is an analysis of the along-grain TS and its reduction coefficient of CBM’s M-L-1, M-L-2, and M-L-3. In Fig 12(a), when the temperature was 100°C, the TS along the grain of M-L-1, M-L-2, and M-L-3 were 29.12 MPa, 33.45 MPa, and 25.26 MPa, which decreased to 0.58 MPa, 2.75 MPa, and 0.24 MPa at 300°C. In Fig 12(b), the TS reduction coefficients of the three specimens along the grain were 0.93, 0.98, and 0.76 at a temperature of 100°C, and significantly decreased to 0.02, 0.05, and 0.01 at a temperature of 300°C. High-temperature would cause serious deterioration of the bonding performance of the interface between the adhesive and the bamboo, making it more likely to cause interface failure when subjected to tensile load, thereby significantly reducing the tensile load-bearing capacity of the composite material.

**Fig 13:**
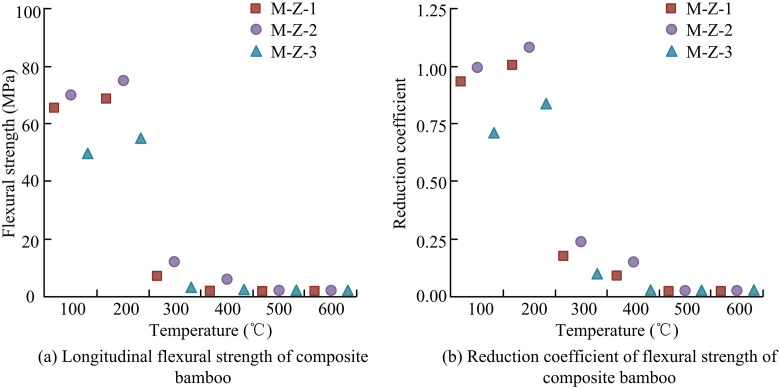
The longitudinal FS and reduction coefficient of composite bamboo.

To explore the influence of different THT on the bending properties of CBM, this study analyzed the grain-following FS and reduction coefficient of M-Z-1, M-Z-2, and M-Z-3 of CBM, as shown in Fig 13. In Fig 13(a), at 100°C, the FS along the grain of M-Z-1, M-Z-2, and M-Z-3 were 64.13 MPa, 72.12 MPa, and 49.75 MPa, and at 300°C they were 7.37 MPa, 12.10 MPa, and 2.52 MPa. In Fig 13(b), the along-grain FS reduction coefficients of the three CBM specimens were 0.93, 1.02, and 0.69 at 100°C, and 0.18, 0.24, and 0.10 at 300°C. As the HTT of MOS adhesive increased, the bending performance of CBM significantly deteriorated, and its ability to withstand complex stress was significantly damaged after high-temperature treatment.

**Fig 14:**
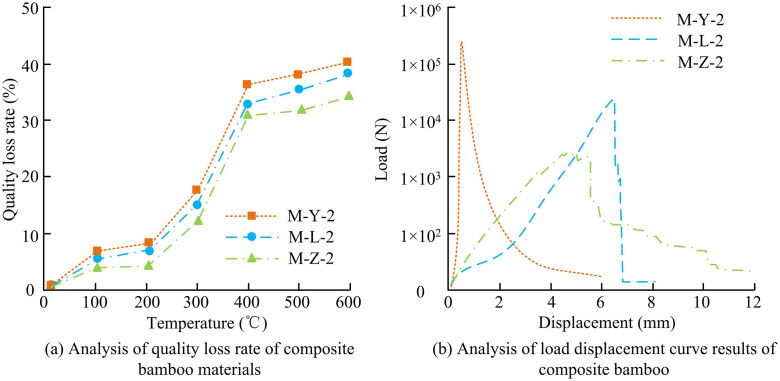
QLR and load displacement curve of composite bamboo.

Fig 14 shows the QLR and load-displacement curves of CBM’s compression specimen M-Y-2, tensile specimen M-L-2, and flexural specimen M-Z-2 after treatment at 200°C. In Fig 14(a), when the temperature is 200°C, the QLRs of M-Y-2, M-L-2 and M-Z-2 were 8.92%, 7.35%, and 3.76%, which increased to 37.12%, 33.28%, and 30.59% at 400°C. This indicated that high-temperature caused a large amount of decomposition of bound water and volatile components in the composite. In Fig 14(b), when the displacement was 0.82 mm, the peak load of the M-Y-2 reached 4.27 × 10^5^ N. When it reached 6.24 mm, the peak load of the M-L-2 was 3.35 × 10^4^ N. When it increased to 4.86 mm, the peak load of the M-Z-2 was 2.67 × 10^3^ N. The compression specimens showed extremely high load-bearing capacity but small deformation, showing brittle failure characteristics. The tensile specimen experienced large plastic deformation before reaching the peak load, showing good ductility. The load-displacement curve of the flexural specimen reflected the progressive failure process of the material under bending stress.

### 3.4. Analysis of FR results of CBM

**Fig 15:**
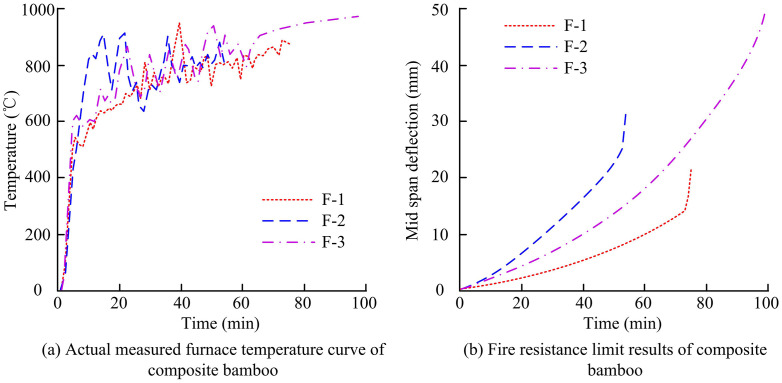
The measured furnace temperature curve and FR limit of composite bamboo.

To explore the FR of the proposed CBM, this study conducted FR tests on CBM specimens prepared by MOS-GA heat-treated at 400°C. This study first analyzed the measured furnace temperature curves and FR limits of CBM’s F-1, F-2, and F-3, as shown in Fig 15. In Fig 15(a), the fire surface temperatures of all specimens rose rapidly at the beginning of the test, reaching approximately 700°C within 10 min, then rising at a slower rate, and finally stabilizing within 950°C-1000°C, in line with the standard fire temperature rise curve. In Fig 15(b), there were obvious differences in the FR times of F-1, F-2, and F-3. The FR time of specimen F-1 was 76 min, and that of specimen F-2 and F-3 was 52 min and 98 min. The maintenance of structural integrity during 98 minutes of fire exposure is primarily attributed to the following microstructural features: the dense 5·1·7 phase network, low pore connectivity, and the formation of a stable MgO/MgSO4 inorganic char layer at elevated temperatures. These features collectively delay heat penetration into the material, inhibit oxygen diffusion, and effectively hinder crack initiation and propagation, thereby ensuring the mechanical integrity and thermal insulation performance of the composite bamboo during prolonged fire exposure. Specimens F-1 and F-3 had better weather resistance, could maintain structural integrity within a certain period, and met the FR requirements of building components.

Table 3 shows the results of the carbonization depth, carbonization rate and internal temperature distribution of CBM specimens under different conditions or time. In Table 3, as the fire exposure time was extended from 20 min to 100 min, the charring depth of all specimens continued to increase, with the F-1 specimen increasing from 5.5 mm to 35.8 mm, F-2 increasing from 4.8 mm to 34.0 mm, and F-3 increasing from 5.3 mm to 35.2 mm. The magnitude of the internal temperature gradient directly reflects the material’s ability to block heat transfer. In this study, when the F-3 component was exposed to fire for 100 minutes, the temperature on the exposed surface was about 950 ° C, while the internal temperature at a distance of 35.2 mm from the exposed surface was only 102 ° C, with a temperature difference of up to 848 ° C and an average temperature gradient of about 24 ° C/mm. This steep gradient indicates that MOS-GA effectively blocks the conduction of heat to the interior. Compared with traditional flame-retardant wood (such as flame-retardant treated laminated wood, which typically has an internal temperature of>200 ° C and a gradient of about 15 ° C/mm after 100 minutes under the same conditions), MOS-GA exhibits better thermal barrier performance due to the endothermic decomposition of inorganic cementitious materials and the formation of dense carbon layers. The internal temperature distribution showed that the F-2 specimen had the highest internal temperature at each time point, while the F-3 specimen had the lowest temperature, showing optimal thermal insulation performance. As the fire exposure time increased, the carbonization depth of CBM increased and the internal temperature distribution increased. Compared to untreated bamboo or polymer-bonded composites, MOS-GA lowers the charring rate of bamboo by forming an endothermic inorganic barrier, which decomposes into MgO and MgSO_4_, consuming heat and delaying thermal degradation.

**Table 3:** Carbonization depth, carbonization rate, and internal temperature distribution of CBMs under different conditions or times.

Exposure Time (min)	Specimen ID	Carbonization Depth (mm)	Carbonization Rate (mm/min)	Internal Temperature (°C)
20	F-1	5.5	0.28	45
	F-2	4.8	0.24	85
	F-3	5.3	0.27	22
40	F-1	13.2	0.33	82
	F-2	12.5	0.31	108
	F-3	12.7	0.32	37
60	F-1	21.0	0.35	113
	F-2	19.8	0.33	136
	F-3	20.7	0.35	61
80	F-1	28.8	0.36	126
	F-2	27.2	0.34	152
	F-3	28.3	0.35	94
100	F-1	35.8	0.36	135
	F-2	34.0	0.34	178
	F-3	35.2	0.35	102

## 4. Discussion

This study explored the effects of different THT on the MP and FR of MOS-GA and its CBM. In the experiment, MOS-GA showed better MP after heat treatment at 400°C, with FS in the range of 9MPa-10MPa and CS in the range of 78MPa-92MPa. As the temperature increased to 600°C, its strength decreased significantly and the microstructure also deteriorated significantly. The decomposition of hydration products led to loose structure and crack expansion. After the MOS-GA’s HTT of CBM increased, its CS, TS, and FS along the grain all showed a downward trend. When the temperature was above 300°C, the strength reduction coefficient decreased significantly. When the HTT was increased to 600°C, its MP decreased due to the decomposition of hydration products, indicating that high temperature causes structural instability. Compared with the reference [6], the MOS-based CBM heat-treated at 400°C in this study performed better in conforming CS, reaching a maximum of 79.36 MPa, which was higher than the strength of its alkali-treated CBM. Reference [14] reported that the CS of MOS materials prepared from solid waste reached 80 MPa, which was equivalent to the strength of MOS-GA treated at 400°C in this study. This further verifies that MOS materials still had excellent mechanical potential at high-temperatures.

In terms of FR, in this study, the FR limit of CBM specimen F-3 using 400°C heat treatment MOS-GA reached 98 min, and the carbonization depth reached 35.2 mm at 100 min, showing good FR thermal insulation performance. This result was better than the structural stability of MOS-reinforced soil at high temperatures in the reference [17], indicating that MOS had better thermal stability and fire-resistant barrier functions in CBM. In addition, the reference [15] used highly active magnesium oxide to improve the coagulation speed and strength of MOS, and its strength was increased by up to 4 times. In this study, heat treatment also enhanced the MP of MOS at moderate temperatures, further verifying the importance of material structure control for performance improvement.

In summary, moderate HTT can effectively optimize the performance of MOS-GA and its CBM, while excessive temperature can lead to structural degradation. Therefore, precise control of HTT is the key to balancing the MP and FR of MOS-based CBM.

## 5. Summary and future work

This study experimentally analyzed the effects of different THT on the MP and FR of MOS-GA and its CBM, and drew the following main conclusions:

(1) MOS-GA had the best MP after heat treatment at 400°C, with CS up to 92.37 MPa and FS in the range of 9 MPa-10 MPa. When the HTT was 600°C, the performance of MOS-GA decreased, the structure became loose, and the hydration products decomposed significantly.
(2) The MP of CBM gradually deteriorated as the HTT of MOS-GA increased. Especially above 300°C, TS and FS decreased significantly, and the interface bonding performance was seriously damaged.
(3) CBM had good comprehensive MP and FR after the MOS-GA was heat treated at 400°C. The CS along the grain could reach up to 79.36 MPa, the FR limit could reach up to 98 min, the carbonization rate was low, and it showed excellent structural integrity and thermal insulation performance.
(4) The FR test showed that the carbonization depth and internal temperature distribution of the CBM specimen under different fire exposure times were reasonable and met the FR design requirements of building components.

Although this study explored the impact of heat treatment on the performance of MOS-based CBM, there are still certain limitations. For example, the HTT range settings are relatively limited and do not cover system comparisons in the medium and low temperature ranges; the long-term durability and moist heat aging performance of CBM have not been evaluated. Therefore, future research will be expanded to multi-scale structural control, dynamic thermomechanical performance analysis and application verification in actual building components to promote the engineering application of this type of composite materials in green FR structures.

## Supporting information

S1 File Minimal data set definition. (DOC)
