# Supplementary material for: Mechanical properties and fire resistance of composite bamboo materials heat-treated with MOS gelling adhesive at different temperatures
Source: PLoS One. 2026 Jun 17;21(6):e0350812. doi: 10.1371/journal.pone.0350812 (PMC13274897; doi:10.1371/journal.pone.0350812)
Supplement: S1 File — (DOC) [file pone.0350812.s001.doc]

**The data in Figure 5**

| Temperature (℃) | Flexural strength (MPa) | | | Flexural strength (MPa) | | |
| --- | --- | --- | --- | --- | --- | --- |
|  | J-1 (7d) | J-2 (7d) | J-3 (7d) | J-1 (28d) | J-2 (28d) | J-3 (28d) |
| 200 | 8.02 | 7.89 | 8.21 | 11.71 | 11.23 | 9.86 |
| 400 | 9.52 | 9.67 | 9.58 | 10.02 | 9.10 | 8.75 |
| 600 | 2.08 | 2.67 | 3.02 | 2.25 | 2.23 | 2.06 |
| 800 | 1.12 | 1.37 | 1.45 | 2.11 | 1.76 | 1.50 |
| 1000 | 0.02 | 0.04 | 0.05 | 0.10 | 0.12 | 0.15 |

**The data in Figure 6**

| Temperature (℃) | Compressive strength (MPa) | | | Compressive strength (MPa) | | |
| --- | --- | --- | --- | --- | --- | --- |
|  | J-1 (7d) | J-2 (7d) | J-3 (7d) | J-1 (28d) | J-2 (28d) | J-3 (28d) |
| 200 | 74.23 | 78.36 | 79.52 | 93.32 | 94.15 | 94.27 |
| 400 | 90.02 | 92.37 | 91.28 | 78.24 | 82.36 | 85.15 |
| 600 | 18.24 | 16.53 | 10.17 | 36.27 | 32.16 | 39.35 |
| 800 | 5.69 | 7.27 | 6.52 | 16.37 | 14.29 | 15.66 |
| 1000 | 0.52 | 0.68 | 0.75 | 0.86 | 0.91 | 0.88 |

**The data in Figure 7**

| Temperature (℃) | Loss on ignition (%) | | | Loss on ignition (%) | | |
| --- | --- | --- | --- | --- | --- | --- |
|  | J-1 (7d) | J-2 (7d) | J-3 (7d) | J-1 (28d) | J-2 (28d) | J-3 (28d) |
| 200 | 9.12 | 8.36 | 8.78 | 5.00 | 5.77 | 6.12 |
| 400 | 14.71 | 15.02 | 15.06 | 14.23 | 13.98 | 12.75 |
| 600 | 19.26 | 18.95 | 18.27 | 16.22 | 16.25 | 17.15 |
| 800 | 20.60 | 21.22 | 21.58 | 17.92 | 18.21 | 18.56 |
| 1000 | 23.76 | 24.02 | 24.21 | 23.65 | 23.51 | 23.42 |

**The data in Figure 11**

| Temperature (℃) | Compressive strength (MPa) | | | Reduction coefficient | | |
| --- | --- | --- | --- | --- | --- | --- |
|  | M-Y-1 | M-Y-2 | M-Y-3 | M-Y-1 | M-Y-2 | M-Y-3 |
| 100 | 39.92 | 42.67 | 33.92 | 0.95 | 1.03 | 0.74 |
| 200 | 68.27 | 79.36 | 50.14 | 1.52 | 1.96 | 1.22 |
| 300 | 59.85 | 60.57 | 48.56 | 0.55 | 0.62 | 0.32 |
| 400 | 6.82 | 9.15 | 3.27 | 0.13 | 0.16 | 0.10 |
| 500 | 4.13 | 4.05 | 1.10 | 0.05 | 0.06 | 0.05 |
| 600 | 0.30 | 0.36 | 0.27 | 0.02 | 0.05 | 0.03 |

**The data in Figure 12**

| Temperature (℃) | Tensile strength (MPa) | | | Reduction coefficient | | |
| --- | --- | --- | --- | --- | --- | --- |
|  | M-L-1 | M-L-2 | M-L-3 | M-L-1 | M-L-2 | M-L-3 |
| 100 | 29.12 | 33.45 | 25.26 | 0.93 | 0.98 | 0.76 |
| 200 | 19.96 | 25.75 | 10.06 | 0.67 | 0.83 | 0.44 |
| 300 | 0.58 | 2.75 | 0.24 | 0.02 | 0.05 | 0.01 |
| 400 | 0.55 | 1.78 | 0.22 | 0.01 | 0.02 | 0.01 |
| 500 | 0.52 | 0.82 | 0.20 | 0.01 | 0.01 | 0.01 |
| 600 | 0.50 | 0.55 | 0.17 | 0.01 | 0.01 | 0.01 |

**The data in Figure 13**

| Temperature (℃) | Flexural strength (MPa) | | | Reduction coefficient | | |
| --- | --- | --- | --- | --- | --- | --- |
|  | M-Z-1 | M-Z-2 | M-Z-3 | M-Z-1 | M-Z-2 | M-Z-3 |
| 100 | 64.13 | 72.12 | 49.75 | 0.93 | 1.02 | 0.69 |
| 200 | 68.91 | 75.36 | 54.15 | 1.04 | 1.11 | 0.83 |
| 300 | 7.37 | 12.10 | 2.52 | 0.18 | 0.24 | 0.10 |
| 400 | 1.98 | 7.21 | 2.40 | 0.12 | 0.16 | 0.07 |
| 500 | 1.67 | 1.72 | 1.70 | 0.07 | 0.05 | 0.05 |
| 600 | 1.55 | 1.61 | 1.58 | 0.05 | 0.05 | 0.05 |

**The data in Figure 14(a)**

| Temperature (℃) | Quality loss rate (%) | | |
| --- | --- | --- | --- |
|  | M-Y-2 | M-L-2 | M-Z-2 |
| 100 | 7.25 | 6.18 | 3.51 |
| 200 | 8.92 | 7.35 | 3.76 |
| 300 | 18.20 | 15.17 | 12.36 |
| 400 | 37.12 | 33.28 | 30.59 |
| 500 | 38.12 | 35.64 | 32.37 |
| 600 | 40.05 | 38.71 | 34.85 |

**The data in Figure 14(b)**

| Displacement (mm) | Load (N) | | |
| --- | --- | --- | --- |
|  | M-Y-2 | M-L-2 | M-Z-2 |
| 2 | 1.78×10^2^ | 685 | 3.65×10^2^ |
| 4 | 372 | 8.12×10^2^ | 2.82×10^3^ |
| 6 | 224 | 1.58×10^4^ | 1.84×10^2^ |
| 8 | / | 121 | 982 |
| 10 | / | / | 665 |
| 12 | / | / | 428 |

**The data in Figure 15**

| Time (min) | Temperature (℃) | | | Mid span deflection (mm) | | |
| --- | --- | --- | --- | --- | --- | --- |
|  | F-1 | F-2 | F-3 | F-1 | F-2 | F-3 |
| 20 | 658.2 | 900.2 | 796.5 | 2.03 | 7.15 | 5.01 |
| 40 | 869.7 | 795.6 | 824.1 | 5.68 | 16.82 | 10.03 |
| 60 | 826.3 | / | 825.2 | 9.98 | / | 18.12 |
| 80 | / | / | 935.6 | / | / | 30.02 |
| 100 | / | / | / | / | / | / |
